# IDEA challenge 2022 dataset: Prototypes from a design Hackathon

**DOI:** 10.1016/j.dib.2024.110363

**Published:** 2024-03-23

**Authors:** Daniel Nygård Ege, Mark Goudswaard, James Gopsill, Ben Hicks, Martin Steinert

**Affiliations:** a Norwegian University of Science and Technology, Richard Birkelandsvei 2B, 7034 Trondheim, Norway; b University of Bristol, Queen's Building, University Walk, Bristol, BS8 1TR, UK

**Keywords:** Design, Prototyping, Product development, Design strategies

## Abstract

The IDEA Challenge 2022 prototyping dataset comprises a total of 240 prototype entries with 1049 edges (connections) and can provide valuable insights into prototyping practices, offering practical knowledge essential for developing prototyping strategies and generating hypotheses for future studies. Data were collected using Pro2booth - an online platform which captured comprehensive information about prototypes and participating teams’ development process, including details about the creators, purpose, timing, and methods of creation. It is particularly relevant to design researchers, engineering and design students, educators, and industry professionals seeking to enhance their prototyping skills and strategies. It serves as a robust foundation for subsequent studies, allowing for comparative analyses, hypothesis verification, and trend exploration. It also has the potential to inform meta-analyses across similar design scenarios, providing a comprehensive understanding of prototyping processes.

Specifications Table

**Table:** 

Subject	Mechanical Engineering
Specific subject area	Prototyping in early-stage design
Type of data	Table Image Chart Graph Figure JSON objects (Text files)
How the data were acquired	Data were acquired from a design study – the IDEA Challenge 2022 – that investigated prototyping design behaviour in a Hackathon. The study utilized Pro2booth, an online platform for tracking prototyping activities. Participants uploaded prototypes to Pro2Booth to capture their prototyping history and design rationale. Pro2booth captures key aspects of each prototype, including the what, who, why, when and how of each. MS Forms was used for questionnaires on demographics, access to prototyping equipment, and self-estimates of expected prototype performance. Physical performance results were derived from tests described in the method section.
Data format	Raw, Analyzed
Description of data collection	Data were generated by 5 teams consisting of PhD students within the engineering design community during a 4- day design hackathon - the IDEA challenge - at their home institutions. Participants uploaded prototypes to Pro2booth throughout the challenge to document their development process. In addition, surveys were conducted to i) understand participant demographics; ii) explore user experience with Pro2booth and the IDEA challenge; and, iii) identify expected design performance during the challenge. Pro2Booth featured mechanisms to check and verify the data before upload.
Data source location	• Institution: Norwegian University of Science and Technology • City/Town/Region:Trondheim, Trøndelag • Country: Norway
Data accessibility	Repository name: zenodo.org Data identification number: 10.5281/zenodo.8328027 Direct URL to data: https://zenodo.org/record/8328027

## 1 Value of the Data

• The IDEA Challenge data offers valuable insights into prototyping practices, informing future strategies and hypothesis generation. This knowledge can guide design education, emphasizing the balance between high-level strategies and operational realities. The data allows for evaluation of different prototyping techniques, thereby driving innovation in design processes and methodologies.
• The dataset would be of particular interest for the following groups: design researchers who can benefit from practical insights into prototyping in rapid innovation scenarios, engineering and design students who can enhance their skills from real-world insights, educators tasked with developing modern curricula, industry professionals looking to improve their prototyping strategies.
• The data can serve as a foundation for subsequent studies in the design field. Other researchers can reuse this data to conduct comparative studies, verify hypotheses, and understand trends. This dataset could also inform meta-analyses across similar hackathons. Besides, the comprehensive understanding of prototyping processes it provides can help researchers in refining methodological approaches for future design studies. Lastly, it can aid in the development of new tools or software for more efficient tracking and analysis of prototyping activities.
• Media files corresponding to prototypes could inform machine learning algorithms, enabling automated analysis and optimization of design features.

## 2 Objective

The primary research objective was to better understand prototyping practices in design teams during rapid innovation events through the capture and subsequent analysis of prototypes generated during a four-day hackathon. By running a design competition that encourages the creation of numerous prototypes, the challenge aims to explore real-world global design challenges and capture valuable insights about design and prototyping activities. This data is then intended to be used by the organizing labs, and potentially other participating institutions, as the basis for joint publications and further research studies.

## 3 Data Description

**Fig. 1:**
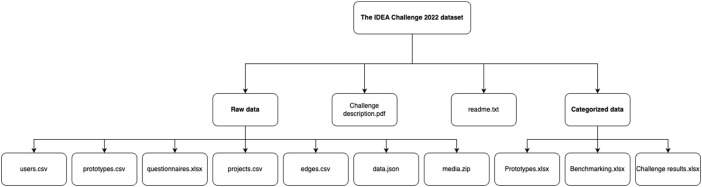
Dataset structure.

The IDEA Challenge dataset [1] contains raw and analyzed data. The diagram in Fig 1. shows how the dataset is structured. A description of individual files and their content is provided in Table 1.

**Table 1:** Dataset content.

	Name	Description
	Readme.txt	Information on the dataset and its structure
	Challenge description.pdf	PDF given to participants at the beginning of the IDEA Challenge. Contains the design brief, expected and required deliverarbles, constraints and assessment criteria
Raw data	Data.json	Data file exported from the online prototype capture tool Pro2Booths database following the IDEA Challenge. Contains prototype upload and corresponding data, connections (creators and influences)
	Media.zip	Zip file containing 349 pictures, videos, CAD files etc. uploaded alongside prototypes in Pro2booth.
	Edges.csv	Contains edges of the dataset (Projects, prototypes, users, connections)
	Projects.csv	Contains the ID of individual teams. Registered users could create PROJECT nodes. These nodes allowed USER and PROTOTYPE nodes to be associated with a specific project. Users linked to a project could upload, edit, and view project prototypes.
	Prototypes.csv	Contains prototypes and their corresponding attribute data. Created by users, these nodes were automatically associated with the active PROJECT node. The PROTOTYPE node includes the following text attributes: • What: Name, description (free text), domain (drop-down menu), media (picture, video, CAD files • Who: Created by • Why: Influences, rationale (free text), purpose according to Camburns’ prototyping purposes [2] (drop-down menu), Insights (free text) • When: Influences, date of capture • How: Method/machinery used (drop-down menu), time to create (drop-down menu)
	Users.csv	Contains the ID of individual participants. Registering as a user in Pro2Booth automatically created a USER node in the database.
	Questionnaires.xlsx	Contains answers to 4 surveys answered by participants: Demographics with relevant experience and education, available prototyping equipment, self-evaluated expected performance, feedback survey
Analyzed data	Prototypes.xlsx	Contains an overview of prototypes sorted by team and a high-level statistical analysis of the dataset, at both team and cohort levels
	Benchmarking.xlsx	Contains results from post-challenge physical performance test of designs.
	Challenge results.xlsx	Contains an overview of how teams were scored based on assessment criteria

## 4 Experimental Design, Materials and Methods

### 4.1 The idea challenge

The International Design Engineering Annual (IDEA) Challenge [[3], [4]] is a virtually hosted hackathon for PhD and post-doctoral researchers in Engineering Design. The objective of running the hackathon was to study prototyping practices, generate open access prototyping datasets, foster community among researchers, and solve real-world design problems.

In the 2022 challenge, participants developed a low-cost portable hydro-power generator to harness rainwater energy. Deliverables included a physical prototype, digital prototypes like CAD models, and a use case scenario. Teams were provided with necessary supplies for comparing and testing designs, described in detail in the “challenge brief” document. The final designs had to be portable and feature custom parts. Teams were given a potential energy limit of 1 kJ for testing their prototypes. Teams had the freedom to decide how to utilize this energy, e.g. suspend a 10 L of water at 10 m or 50 L of water at 2 m.

The challenge saw participation from five teams from universities across Europe, each consisting of 3–4 participants. Participants were invited to take part in the study on the basis of their roles as researchers within the engineering design community at various European institutions, ensuring expertise and active involvement in this field. Contributing to the study and the dataset was a prerequisite to participate. This was stipulated in the consent form that each participant signed as part of the study's ethics procedure. The participants, aged between 23 and 35 years, were primarily PhD students, with one post-doc. Most participants were from the field of mechanical engineering, with others from industrial design, aerospace engineering, and computer science. Their design experience ranged from less than two to over ten years. Complete demographics for each participant include age, gender, the participants team number, current academic position, design experience, specific fields of expertise and practical industry experience, and can be found in the questionnaire.xlsx file in the dataset. Participants contributed from their respective home institutions, where they had ready access to familiar tools and technologies. A list of available tools and equipment available to each team for duration of the hackathon is provided in the Questionnaires.xlsx file.

### 4.2 Data capture method - Pro2booth

**Fig. 2:**
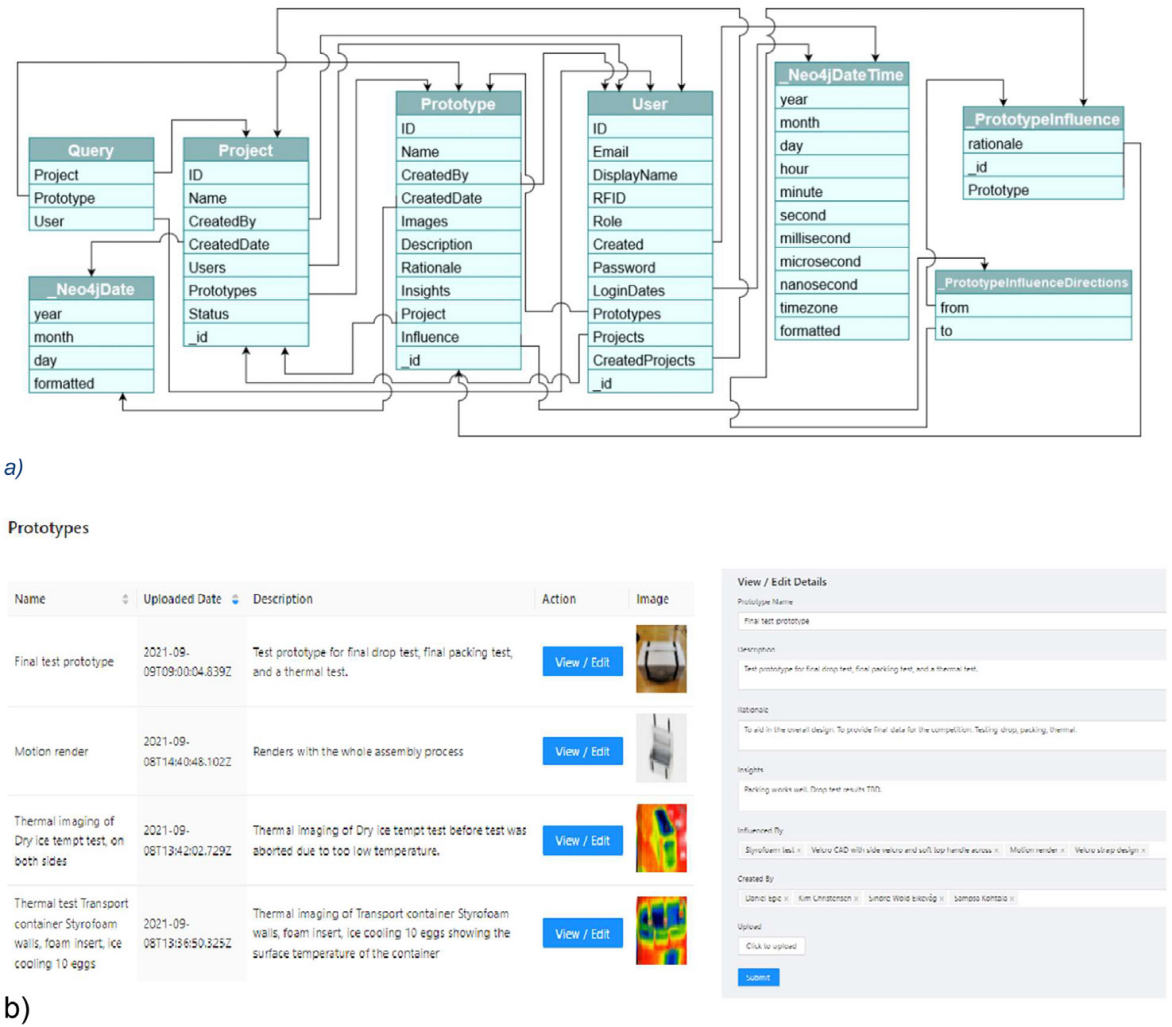
a) Graph database schema and b) User interface (from [6]).

Pro2booth is an online system designed to capture and track the development of prototypes during a design process [5]. It's a web-app that encourages participants to document their prototypes in real-time. The system works by storing data in a Graph Database where the nodes were users, prototypes and projects. Edges were used to link users to projects and prototypes and prototypes to projects and other prototypes. Attributes were captured on the nodes and edges and media was captured in a blob store and referenced in the database. The graph database schema is shown in Fig. 2a and the user interface in 2b.

During the IDEA Challenge, participants were motivated to utilize Pro2booth consistently. The competition's scoring system was designed to reward teams based on their usage of the platform. The number of prototypes and the quality or completeness of the entries determined the points each team earned on a daily basis. Participants were given a thorough introduction to the system and definitions used to ensure comparability.

Certain fields were auto-populated, such as Dates and IDs. Users manually entered other details. The following aspects of each prototype are captured by Pro2booth and can be arranged into the **what, who why, when** and **how** of prototyping:

• **What:** Name, description (free text), domain (drop-down menu), media (picture, video, CAD files
• **Who:** Created by
• **Why:** Influences, rationale (free text), purpose according to Camburns’ prototyping purposes [2] (drop-down menu), Insights (free text)
• **When:** Influences, date of capture
• **How:** Method/machinery used (drop-down menu), time to create (drop-down menu)

Data for each attribute was mandatory for a prototype to be accepted and created in the database. This requirement facilitated the transfer of knowledge from the design team into the application. Additionally, the user creating the prototype was required to submit media content, such as images and videos, for reference. The user did not need to be directly involved in the prototyping activity, as they could reference other users involved. Prototype attributes could be edited and updated post-creation, until the conclusion of the hackathon.

One of the key features of Pro2booth is its ability to capture the interrelationships between prototypes. This means it can track how different prototypes influence each other during the design process. Pro2booth also connects the creators of each prototype. This provides a clear record of who contributed to what, making it a valuable tool for collaboration and accountability.

The database of prototypes was exported as a file named 'data.json' in the dataset. Accompanying media such as pictures and videos were stored in a 'media.zip' file. CSV files containing edges, prototypes, projects, and users were generated by querying these fields within the 'data.json' file.

Prior to commencing the hackathon, participants completed questionnaires pertaining to demographics and the availability of prototyping equipment. On the end of day 2 and the end of day 3, self-estimations regarding the expected performance of the final designs were collected. Finally, after the final presentations on day 4, participants responded to a feedback survey. All questionnaire answers are reported in the ‘questionnaires.xlsx’ file.

### 4.3 Anonymisation

Names and team names have been replaced with consistent alternative values to preserve the integrity of the dataset. Some media were omitted upon request and are indicated in the graph with the 'XX' label.

### 4.4 Benchmarking

Following the conclusion of the IDEA Challenge, the design prototypes were shipped to the organizers to undergo an objective benchmarking and testing process. The setup for all tests remained constant. An Adafruit 12 V servo motor was attached to each prototype by means of a shaft coupler, and a rectifying circuit and an Adafruit INA260 power meter connected to an Arduino Uno were deployed to compute the output power.

A garden hose (ID=13 mm) and the same nozzle was used across testing each design. A water reservoir containing 5 L of water was suspended 10 m above the prototypes, giving E_p_=mgh=5 × 10×10=500 J. Emptying the water tank took 58 s, giving P_pot_=8,57 W. The power output of prototypes during testing was compared to P_pot_ to determine efficiency in%.

## Limitations

Prototype entries were self-reported with the risk of subjective interpretation of terms and definitions. To mitigate this risk we provided a thorough introduction to the Pro2booth platform and definitions used to ensure comparable results. The resources available to each team varied based on their research group environments, which means the prototyping practices observed might reflect specific resource availability rather than general practices. The IDEA Challenge's focus on the number of prototypes as part of its incentivization structure might have led teams to prioritize quantity over quality, potentially skewing the data. Finally, the unique context of a hackathon may not fully reflect the broader spectrum of design and prototyping processes in different settings, although sharing multiple characterizations.

## Ethics statements

Informed, written consent was obtained from all participants of the study. The study was granted ethical clearance for Research Data by NSD/SIKT, ref. 226091 (2022-04-26).

## CRediT authorship contribution statement

**Daniel Nygård Ege:** Conceptualization, Methodology, Validation, Investigation, Data curation, Writing – original draft, Writing – review & editing, Visualization, Project administration. **Mark Goudswaard:** Conceptualization, Methodology, Validation, Investigation, Writing – review & editing. **James Gopsill:** Methodology, Conceptualization, Writing – review & editing. **Ben Hicks:** Conceptualization, Methodology, Supervision. **Martin Steinert:** Conceptualization, Methodology, Supervision.

## Data Availability

IDEA Challenge 2022 dataset (Original data) (Zenodo).
